# Prescribing and monitoring high dose and combined antipsychotics in community mental health team

**DOI:** 10.1192/bjo.2021.476

**Published:** 2021-06-18

**Authors:** Dipanjan Banerjee, Tracey Green, Umama Khan

**Affiliations:** 1 East Sussex Healthcare NHS Trust; 2 St Marys Hospital; 3 St Marys Hospital

## Abstract

**Aims:**

The aim was to establish the prevalence of prescription of combined and high dose antipsychotics in the community mental health team and to see if such patients were being offered regular monitoring as advised by NICE guidelines.

**Background:**

The use of high dose antipsychotic treatment (HDAT) should be in line with the recommendations of the Royal College of Psychiatrists Consensus statement on high dose antipsychotic Treatment. Such treatment should be initiated only when standard treatments have failed. As high dose prescribing of antipsychotic medications can potentially harm than benefit causing serious side effects and sudden death. Close monitoring and documentation are required. Also, reviewing these patients at regular intervals is recommended.

**Method:**

A retrospective audit of 50 case notes of patients currently on antipsychotics was done. Case notes were selected randomly from a pool of 300 plus patients under the care of Isle of Wight NHS Trust in the HoNos Cluster 11 and 12 care pathways. Data were gathered on patients` demographics, diagnosis, medication monitoring. Data were analysed and discussed with consultant psychiatrist and senior mental health pharmacist. Patients who were not on any antipsychotic medications were not included in the audit.

**Result:**

90% of the patients were on single antipsychotic (45 out of 50), 4.45% (2 out of 45)were above BNF recommended dose. 10% (5) patients were prescribed combined antipsychotics. 40% (2)of them were above BNF recommended maximum dose. A total of 8% (4 out of 50) patients were on above BNF recommended maximum dose. All the patients on high dose antipsychotics had a clear plan documented in the system. Documented monitoring of Full blood count was found in 75%, blood glucose in 50%, lipid in 75%, cardiac monitoring (Electrocardiogram or ECG) in 0%, physical health monitoring in 0%

**Conclusion:**

As far as we know, this is the first time an audit has been done on the patients under the Community Mental Health Team on high dose antipsychotic treatment. The data showed areas of good practice as the majority of the patients were on monotherapy, and all the patients on a high dose or combined antipsychotics had clear management plans outlined in their notes. However, the audit also highlighted areas that currently need improvements such as regular monitoring of the patients on high dose and combined antipsychotics. It should also be clearly documented, recorded, and reviewed at six-monthly intervals.

